# Differences and Similarities between Colorectal Cancer Cells and Colorectal Cancer Stem Cells: Molecular Insights and Implications

**DOI:** 10.1021/acsomega.3c02681

**Published:** 2023-08-09

**Authors:** Derya Erisik, Berrin Ozdil, Eda Acikgoz, Cemile Sinem Asker Abdikan, Taha Kadir Yesin, Huseyin Aktug

**Affiliations:** † Department of Histology and Embryology, Faculty of Medicine, Ege University, Izmir 35100, Turkey; ‡ Department of Histology and Embryology, Faculty of Medicine, Suleyman Demirel University, Isparta 32260, Turkey; § Department of Histology and Embryology, Faculty of Medicine, Yuzuncu Yil University, Van 65080, Turkey

## Abstract

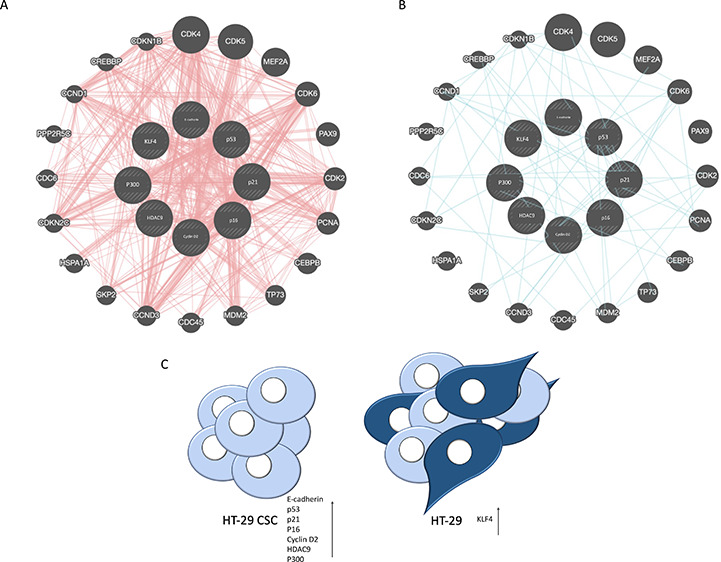

Malignant tumors are formed by diverse groups of cancer cells. Cancer stem cells (CSCs) are a subpopulation of heterogeneous cells identified in tumors that have the ability to self-renew and differentiate. Colorectal cancer (CRC), the third most frequent malignant tumor, is progressively being supported by evidence suggesting that CSCs are crucial in cancer development. We aim to identify molecular differences between CRC cells and CRC CSCs, as well as the effects of those differences on cell behavior in terms of migration, EMT, pluripotency, morphology, cell cycle/control, and epigenetic characteristics. The HT-29 cell line (human colorectal adenocarcinoma) and HT-29 CSCs (HT-29 CD133^+^/CD44^+^ cells) were cultured for 72 h. The levels of E-cadherin, KLF4, p53, p21, p16, cyclin D2, HDAC9, and P300 protein expression were determined using immunohistochemistry staining. The migration of cells was assessed by employing the scratch assay technique. Additionally, the scanning electron microscopy method was used to examine the morphological features of the cells, and their peripheral/central elemental ratios were compared with the help of EDS. Furthermore, a Muse cell cycle kit was utilized to determine the cell cycle analysis. The HT-29 CSC group exhibited high levels of expression for E-cadherin, p53, p21, p16, cyclin D2, HDAC9, and P300, whereas KLF4 was found to be high in the HT-29. The two groups did not exhibit any statistically significant differences in the percentages of cell cycle phases. The identification of specific CSC characteristics will allow for earlier cancer detection and the development of more effective precision oncology options.

## 1 Introduction

CRC, which encompasses colon and rectal cancer (ICD-10 positions C18–C20),^1^ is the third most prevalent malignant tumor around the world.^2^ High-income countries’ aging populations, male gender, diet, obesity, sedentary lifestyle, and smoking are all CRC risk factors.^3^ The main forms of treatment for cancer patients have been surgery and chemotherapy, but the prognosis for CRC, especially in patients with metastatic lesions, has never been promising. A new optional approach called targeted therapy has significantly enhanced the CRC patients’ general survival.^4^

According to new research, only a phenotypic group of the tumor-forming cancer cell population can initiate tumor growth in various human malignancies.^5,6^ This functional and minority subpopulation of cancer cells is termed CSCs that are distinguished by their ability to regenerate themselves, proliferate indefinitely, and generate tumor cells with a more differentiated phenotype.^7,8^ CSCs are more malignant than other tumor-forming cells, as they have important roles in cancer initiation, increased invasion, spread of cancer, and resistance to conventional cancer treatments. Due to the described characteristics, it is emphasized that CSCs are very important in determining the clinical behavior of tumors and should be the focus for developing better therapies.^9^

There is increasing evidence that CSCs exist in human CRC.^5,10^ CRC CSC populations have been effectively separated from human colon tumors using the CD133 surface marker in studies,^10,11^ and the tumorigenic potential appeared solely in the CD133^+^ cell population, whereas the CD133^–^ cells showed no such effect.^11^ Understanding the role of colon cancer-forming CSCs in tumor initiation and progression can be enhanced by correctly characterizing CSCs.

Epigenetics refers to epi-information that exists beyond the basic sequence of DNA, is inherited, and can be passed down from generation to generation.^12^ Histone acetylation is a well-known epigenetic mechanism that involves the acetylation by HATs and deacetylation of histones by HDACs, which regulates gene activity.^13^ Elevated HDAC levels are frequently linked to disease progression and poor clinical outcomes in patients.^14^ Additionally, the type of tissue determines which HDAC is in charge of the malignant cancer cell behavior. The involvement of HATs, one of which is P300, in tumor growth and metastasis remains significant.^15^

HDAC proteins have a role in gene activity in the course of the cell cycle.^16^ Cyclins and cyclin-dependent kinases (Cdks) serve as essential cell cycle regulators, and Cdk inhibitors as p21 modulate the activity of Cdks.^17^ Cells have control systems known as checkpoints that regulate the cell cycle from one phase to the next and prevent cycle progression response to replication stress or DNA damage.^18^ Checkpoint proteins, such as p53, are phosphorylated when the checkpoint pathway is activated, resulting in activation.^19,20^ HDACs deacetylate and regulate the activities of important cell cycle proteins such as p53, leading to neoplastic transformation.^21^ Few studies have looked at the connection between cell cycle dysregulation and CSC traits hypothesized to be responsible for tumor initiation despite the growing attention in CSCs.^22,23^

Downregulation of CDH1, a marker for epithelial cells also known as epithelial-cadherin, is a defining feature of cancer metastasis. Depletion of E-cadherin helps in the epithelial-mesenchymal transition (EMT).^24,25^ Tight junction complexes, cell polarity, and the cytoskeleton of epithelial tumor cells are lost during EMT, resulting in more invasive capabilities and phenotypes.^26^

According to numerous studies, CSCs are associated with tumor invasion, high metastatic potential,^27^ and resistance induced by radiotherapy or chemotherapy.^28,29^ Networks of transition states are linked to pluripotency and CSCs.^30^ Krüppel-like factor 4 (KLF4) acts in pluripotency. The extent to which CSCs have pluripotent properties and whether this leads to a hierarchical structure among cancer cells is a topic that is frequently discussed today.

The aim of our research is to reveal the molecular differences between CRC cells and CSCs and to understand the effects of existing differences on cell behavior in terms of migration, EMT, pluripotency, morphology, cell cycle/control, and epigenetics characteristics.

## 2 Materials and Methods

### 2.1 Conditions and Reagents for Cell Culture

The HT-29 (ATCC; HTB-81) cell line was used to respresent human colorectal cancer. The cells were cultured in RPMI-1640 (Sigma-Aldrich) supplemented with 10% FBS (Gibco, Invitrogen Life Technologies) with 1% penicillin/streptomycin (Sigma-Aldrich) and incubated at 37 °C in 5% CO_2_. Under an inverted microscope, cells were examined daily for general cellular morphology, proliferation, and contamination. Cells were diluted to 2 × 10^5^ for experimental use.

### 2.2 CD133^+^/CD44^+^ Colorectal CSC Isolation and Culture Conditions

Flow cytometry was used to isolate the CD133^+^/CD44^+^ cells in the HT-29 cell line. A total of 1 × 10^6^ cells/mL were labeled by the incubation with 10 μL of CD133-PE and CD44-APC (Miltenyi Biotec, Bergisch Gladbach) for 10 min at 4 °C. The CD133^+^/CD44^+^ subpopulation was then filtered and isolated by a FACSAria II flow cytometer for CD133^+^/CD44^+^ markers to gain HT-29 CSCs.^31^ HT-29 CSCs were grown in HT-29 cell culture conditions.

### 2.3 Staining for Immunohistochemistry

HT-29 and HT-29 CSCs were diluted to 2 × 10^5^ cells/mL and cultured at 37 °C 5% CO_2_ for 72 h. At room temperature, the samples were fixed with 4% PFA for half-hour. They were then permeabilized with 0.25% Triton X-100 for 10 min. After that, the samples were blocked for an hour with 3% BSA. Primary antibodies E-cadherin (Santa Cruz Biotechnology, H3109), KLF-4 (Invitrogen, TL275284), p53 (Bioss, 9H20V16), p21 (Bioss, BB07142233), p16 (Bioss, BB10114929), cyclin D2 (Santa Cruz Biotechnology, F2706), HDAC9 (Bioss, BA11018514), and P300 (Bioss, 9I22V41) were diluted at 1/200. Cells were incubated for one hour with a secondary antibody (SHP125 from ScyTek Laboratories). It was then coated with HRP-streptavidin solution and rinsed with PBS. The final coloring was elucidated by DAB solution (ScyTek Laboratories, ACK125) and hematoxylin (Harris, Merck). The images were captured with an Olympus BX51 microscope (Olympus Optical Co., Tokyo, Japan).

### 2.4 Scratch Assay

We used a scratch assay to determine the migration capacity of HT-29 cells and HT-29 CSCs. After passage and dilution to 2 × 10^5^ cells/mL, cells were seeded to form a confluent monolayer. In proper incubation conditions, cells were allowed to completely adhere and confluence for 72 h. By manually scraping with a 200 μL pipette, a wound was formed. The initial image was captured with the reference of the scratch on the plate. Images were taken at 0, 24, 48, and 72 h, and the distance migrated by the cells at the leading edge was measured by taking three measurements at predetermined points in each well. Each experimental point was carried out in triplicate.

### 2.5 Scanning Electron Microscopy/Energy-Dispersive X-ray Spectroscopy (SEM/EDS)

HT-29 and HT-29 CSCs were diluted to 2 × 10^5^ cells/mL and cultured at 37 °C 5% CO_2_ for 72 h. To fix the samples, they were treated with 4% PFA and kept in PBS. Before SEM/EDS analysis, specimens were thoroughly dried with air. All specimens were covered with 6 nm gold–palladium in a Leica EM ACE600 (Leica Microsystems, Germany) sputter coater under vacuum. With argon gas, this process took about 30 min. The acceleration voltage used for imaging was 1 kV. 0.9 nm was the photographic resolution. A Thermo Scientific Apreo S LoVac SEM (Thermo Fisher Scientific, USA) with a Schottky field emission gun was used for SEM/EDS imaging. The images were obtained in the high vacuum mode, and for the measurements, a maximum beam current of 50 nA and an accelerating potential of 30 kV were utilized. An EDAX instrument from AMETEK, USA, was used in the experiments to analyze the elemental spectra from each cell. The working distance for the samples was 10.4 mm for HT-29 and 10.5 mm for HT-29 CSCs. An accelerating voltage of 5 kV was applied across all cells to generate the electron beams. HT-29 and HT-29 CSCs were imaged at 10,000–15,000×.^32^

### 2.6 Cell Cycle Analysis

HT-29 and HT-29 CSCs were passaged and diluted to 2 × 10^5^ cells/mL and incubated for 72 h at 37 °C in 5% CO_2_. After passage, cells were fixed in ice-cold ethanol overnight. Following that, cells were rinsed with 1× PBS and incubated for half-hour with a Muse cell cycle reagent kit. A Muse cell analyzer was used to display cell cycle phases.

### 2.7 Statistical Analysis

The cell cycle, scratch assay, and comparisons of protein expression were evaluated by IBM SPSS Statistics 25.0. For protein expression, H-score was performed. At least one hundred cells in each cell group were counted blindly by three different histologists, and the protein expression percentages were compared. The data were analyzed by normality testing using the Shapiro–Wilk test and variance homogeneity testing using Levene’s test. The data distribution was not normal; therefore, analyses were performed with the Mann–Whitney-U test. Unless otherwise specified, results were presented as the mean standard error (SE). The statistically significant difference was defined as *p* < 0.05.

## 3 Results

**Figure 1:**
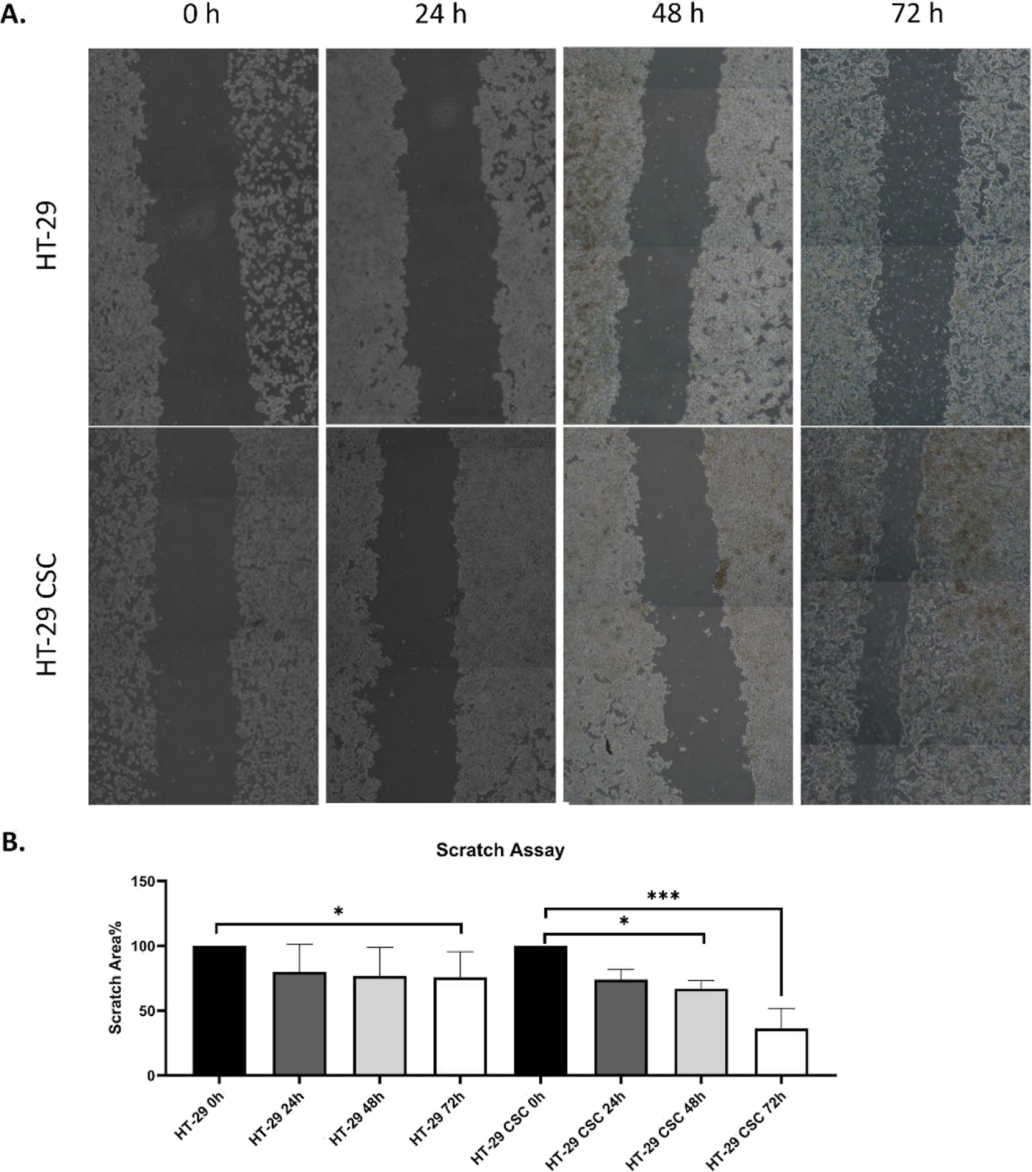
(A) Representative scratch assay images showing the ability of HT-29 and HT-29 CSCs to migrate. Scale bar, 20 μm. (B) Graph for the scratch assay of HT-29 and HT-29 CSCs. The asterisks (*) represent significant (*p < 0.05, ***p < 0.001) differences between groups. While the HT-29 scratch assay was significant only at 72 h, HT-29 CSC cells showed a more rapid and significant closure potential. Area closure was statistically different in HT-29 CSCs at 48 and 72 h in comparisons (respectively *p < 0.05 and ***p < 0.001), while the difference was only significant at 72 h in the HT-29 cell group (*p < 0.05).

Migration of both HT-29 and HT-29 CSCs was quantified by the ability of cells to move into the acellular space by the scratch assay. We compared the cell migration potential in HT-29 and HT-29 CSCs at 0, 24, 48, and 72 h, and findings showed the 0 (100; 100), 24 (80; 74), 48 (76.9; 67), and 72 h (75.6; 36) percentages of cells in HT-29 and HT-29 CSCs (Figure 1). The HT-29 scratch assay was significant at only 72 h, while HT-29 CSCs showed a faster and more significant area closure potential at 48 and 72 h. The scratch assay indicates that while the migration capacity was similar between cancer and CSCs, the latter exhibited a higher tendency for closure, suggesting greater activity in the closure.

**Figure 2:**
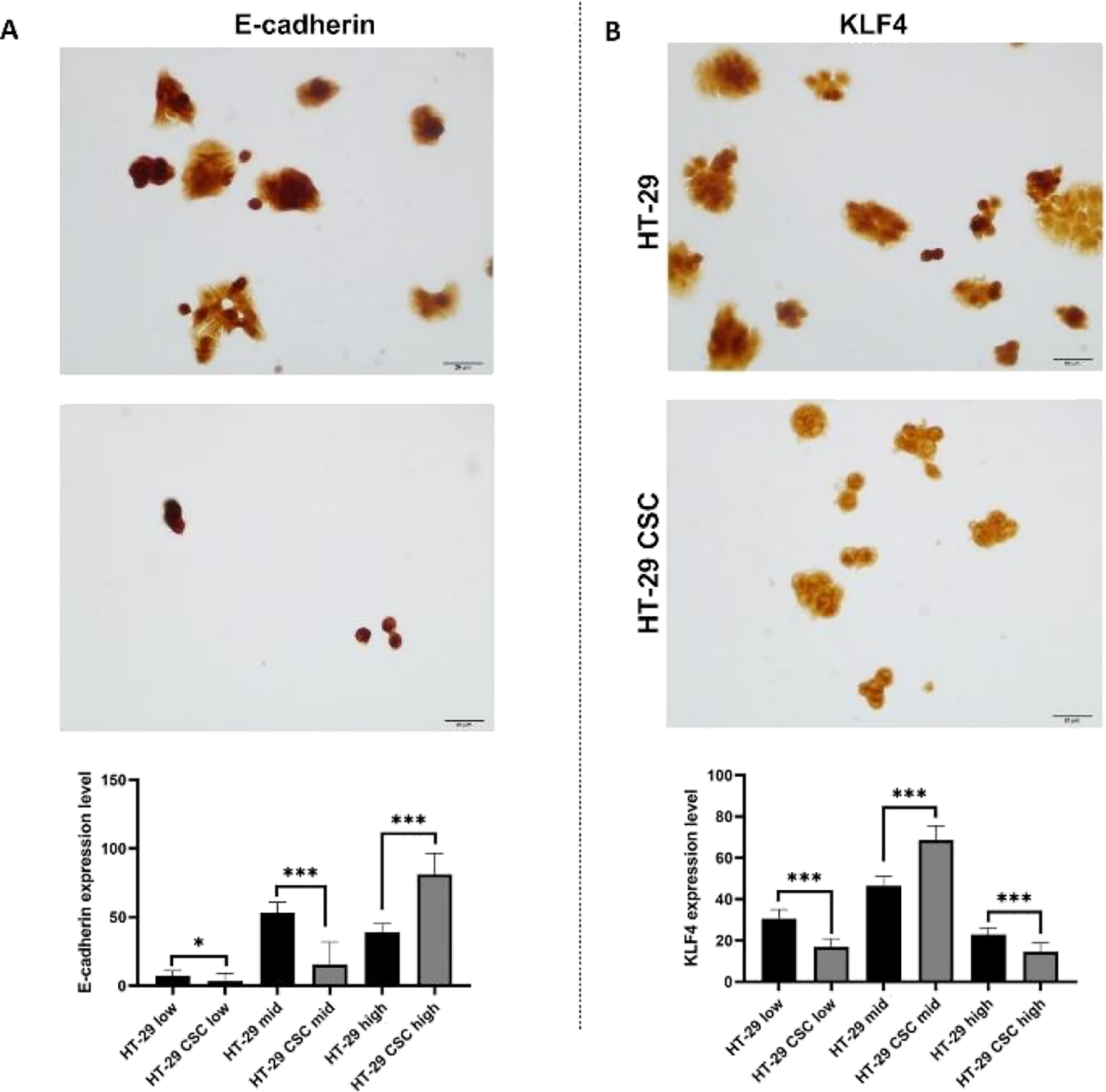
Immunocytochemistry images and graphs of KLF4 and E-cadherin expression levels of HT-29 and HT-29 CSCs. Scale bar, 20 μm. Protein expressions were assessed using a histopathological scoring system. The asterisks (*) represent significant (*p < 0.05, ***p < 0.001) differences between HT-29 and HT-29 CSCs. (A) Low expression of E-cadherin protein that was statistically higher in the HT-29 group (*p < 0.05) than HT-29 CSCs. E-cadherin protein was higher in the mid-expression HT-29 group (***p < 0.001), while it was elevated in high-expression E-cadherin protein in HT-29 CSCs (***p < 0.001). (B) Low expression of KLF4 protein that was statistically higher in the HT-29 group (***p < 0.001) than HT-29 CSCs. The KLF4 protein level was higher in the mid-expression HT-29 CSC group (***p < 0.001), while it was elevated in the high-expression KLF4 protein level in HT-29 (***p < 0.001).

We compared E-cadherin protein levels in HT-29 cells and HT-29 CSCs in our research, and findings show the low-expression (7.4593 ± 1.23155; 3.4623 ± 1.40976), mid-expression (53.3556 ± 2.64938; 15.5290 ± 4.10309), and high-expression (39.1850 ± 2.17315; 81.0087 ± 3.88614) levels of cells in HT-29 and HT-29 CSCs, respectively. High expression of E-cadherin protein was higher in the HT-29 CSC group (Figure 2A). These results showed that the upregulation of the cell–cell adhesion molecule E-cadherin in CSCs could be a notion of the tumor-promoting role.

We compared KLF4 protein levels in HT-29 cells and HT-29 CSCs in our research, and findings show the low-expression (30.5770 ± 1.31966; 16.8593 ± 1.23281), mid-expression (46.5312 ± 1.36191; 68.5655 ± 2.16874), and high-expression (22.8919 ± 0.91350; 14.5753 ± 1.32124) levels of cells in HT-29 and HT-29 CSCs, respectively. High expression of KLF4 protein was higher in the HT-29 group (Figure 2B). Here, the zinc finger transcription factor KLF4 was downregulated in CSCs compared to HT-29 cell lines.

**Figure 3:**
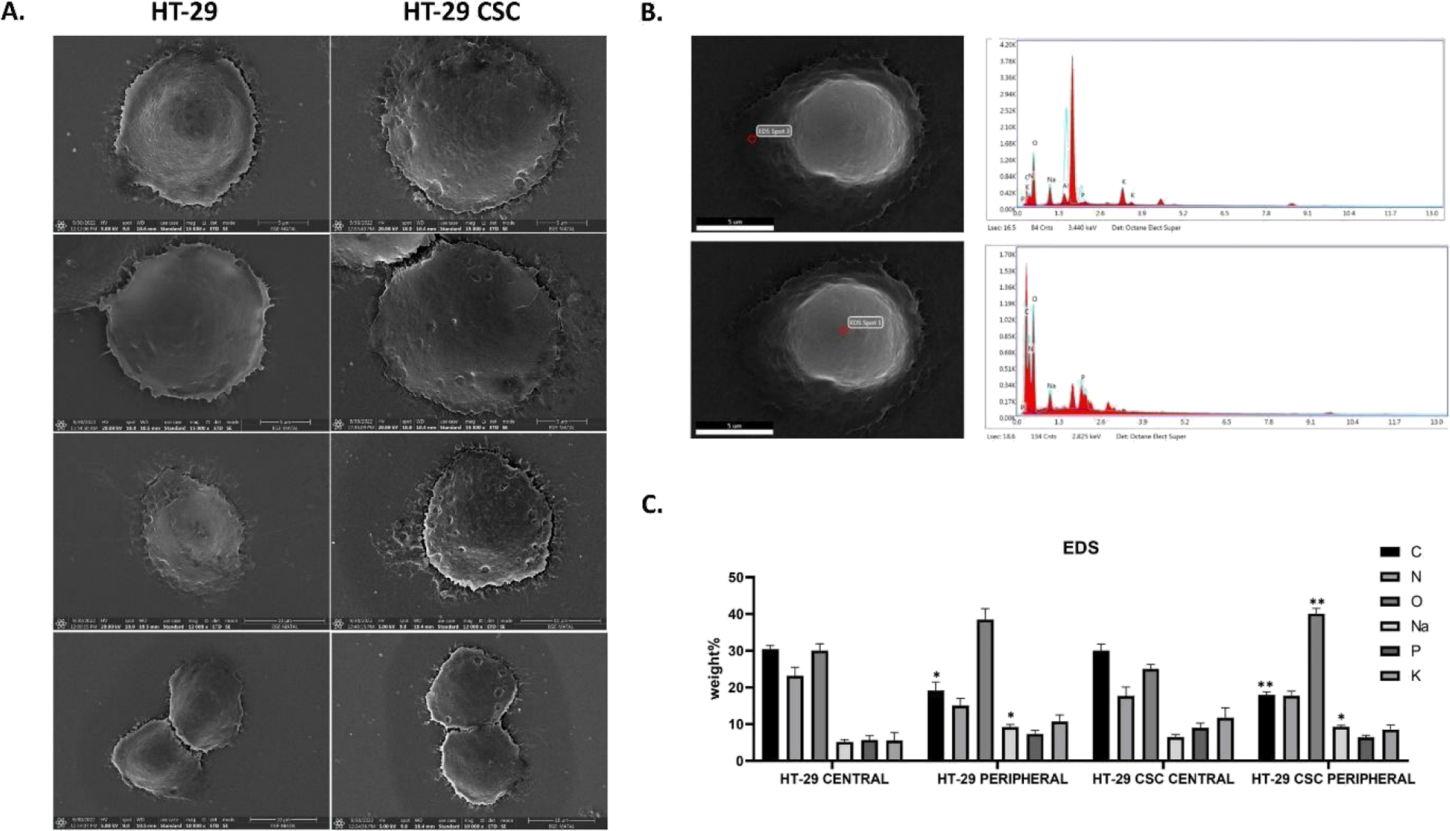
HT-29 and HT-29 CSCs SEM/EDS images. (A) SEM images of the HT-29 and HT29 CSCs. These cells were imaged at 10,000, 12,000, and 15,000×. (B) Representative central and peripheral areas of interest (spot) for EDS analysis and representative EDS spot analysis spectral graphs. (C) Mean weight elemental percentages of HT-29 and HT-29 CSCs’ central and peripheral areas. These cells were compared with SEM/EDS, and six different elemental signals were obtained. Weight (%) results were represented as the sample mean values and evaluated as semiquantitative data. The asterisks (*) represent significant (*p < 0.05, **p < 0.01) differences between HT-29 and HT-29 CSCs. The mean weight percent of the element C of HT-29 is higher in the central area than in the peripheral area (*p < 0.05). The mean weight percent of the element Na of HT-29 is lower in the central area than in the peripheral area (*p < 0.05). The mean weight percent of the element C of HT-29 CSCs is higher in the central area than in the peripheral area (**p < 0.01). The mean weight percent of the element O of HT-29 CSCs is lower in the central area than in the peripheral area (**p < 0.01). The mean weight percent of the element Na of HT-29 CSC is lower in the central area than in the peripheral area (*p < 0.05).

A significant amount of control over cellular processes is provided by the cell morphological profile, which also represents cell identity. It can therefore be thought of as the key to the system that controls cell fate and processes. The ultrastructures of HT-29 and HT-29 CSCs were well-documented in this research using SEM. We compared the morphological features of HT-29 and HT-29 CSCs, which are displayed in Figure 3. As seen from the SEM results, HT-29 and HT-29 stem cells morphologically tend to form colonies. The cells had irregular borders and numerous finger-like projections of the plasma membrane of varying lengths. According to our SEM results, the filopodia number of HT-29 cells was 8.1429 ± 1.81827 per cell, the filopodia length was 2.7315 ± 0.26932 μm, the filopodia number of HT-29 CSCs was 5.0833 ± 0.80206 per cell, and the filopodia length was 2.0175 ± 0.11017 μm. The cells had central (nuclear) and peripheral areas, with a distinct lamellipodial area at the periphery. Several spots from the central and peripheral areas were analyzed for elemental ratio comparison during SEM/EDS analysis. According to the EDS results, the most representative elements discovered were carbon (C), nitrogen (N), oxygen (O), sodium (Na), phosphorus (P), and potassium (K). The mean weight percentages of the element C of HT-29 cells and HT-29 CSCs were higher in the central area than in the peripheral area (*p < 0.05, **p < 0.01). The mean weight percentages of the element Na of HT-29 cells and HT-29 CSCs were lower in the central area than in the peripheral area (*p < 0.05). The mean weight percent of the element O of HT-29 CSCs was lower in the central area than in the peripheral area (**p < 0.01). When the distribution percentages of other elements (N, P, and K) in the peripheral and central areas were compared, there was no significant difference (*p* > 0.05). A comparison of elemental ratios reveals regional differences in cancer and CSCs based on central and peripheral areas.

**Figure 4:**
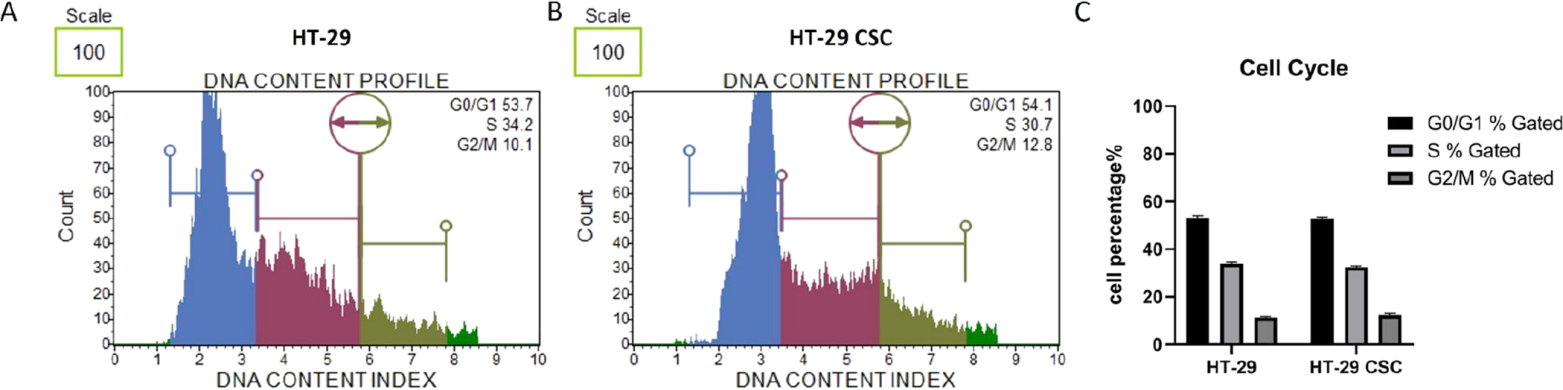
Cell cycle analysis of HT-29 cells and HT-29 CSCs. These cells were incubated for 72 h. A Muse cell cycle kit was used to determine the percentages of G0/G1, S, and G2/M phases and compare them. (A) HT-29 graph example. (B) HT-29 CSC graph example. (C) Cell cycle distribution histogram representing the percentages of cells in the G0/G1, S, and G2/M phases. HT-29 cells and HT-CSCs were found in the cell cycle phases of G0/G1 (53.0; 52.7), S (34.0; 32.5), and G2/M (11.4; 12.4), respectively. This graph displays the results of three independent experiments. There was no significant difference in cell cycle analysis results between HT-29 and HT-29 CSCs (*p* > 0.05).

We compared cell cycle phases in HT-29 cells and HT-29 CSCs, and findings show the G0/G1 (53.0; 52.7), S (34.0; 32.5), and G2/M (11.4; 12.4) percentages of cells in HT-29 and HT-29 CSCs, respectively (Figure 4). According to the results of cell cycle analysis, no significant difference was found between the groups (*p* > 0.05). The cell cycle analysis revealed that the percentage of cells in each cell cycle phase was comparable between the cancer and CSC groups.

**Figure 5:**
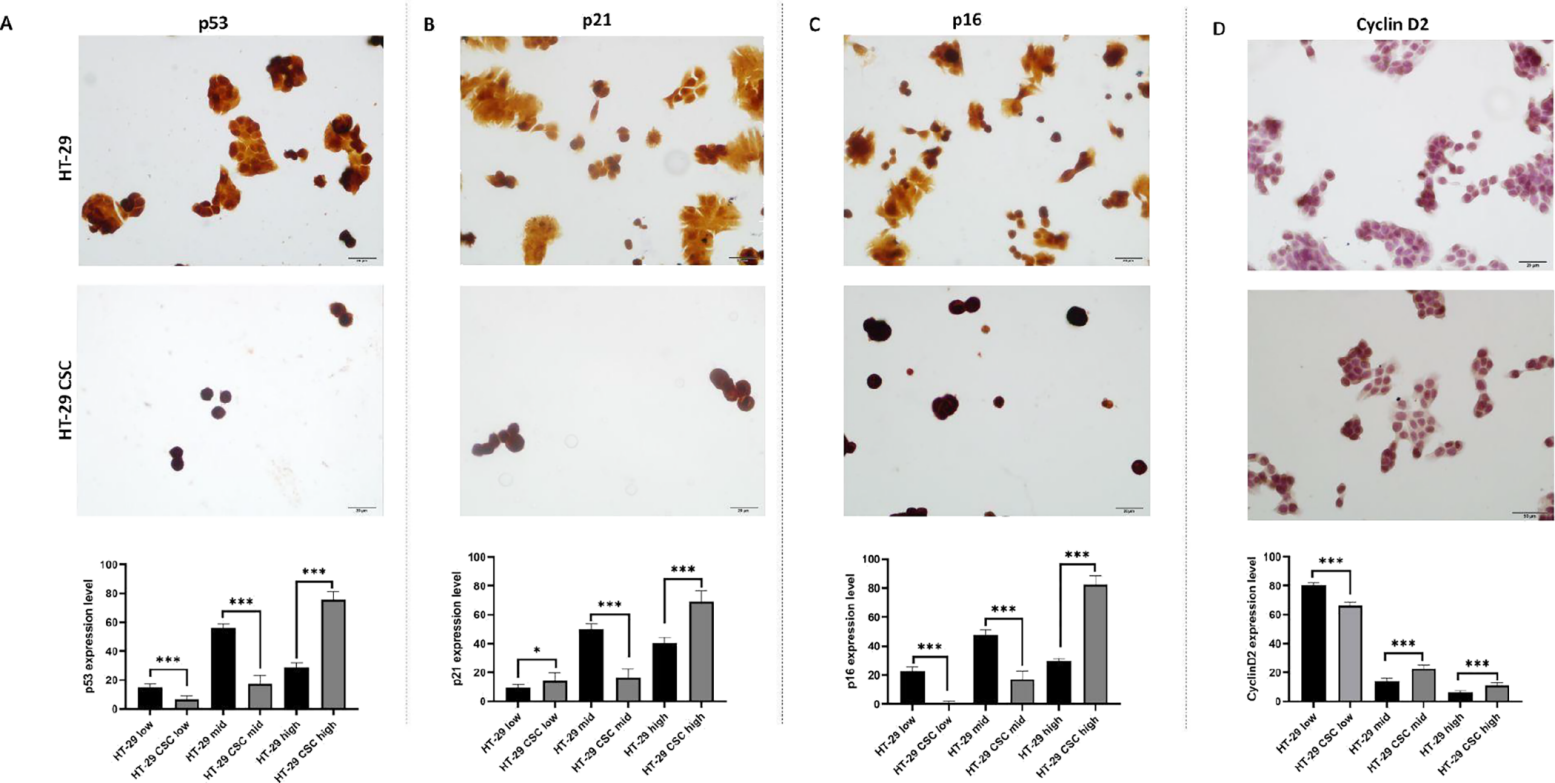
Immunocytochemistry images and graphs of p53, p21, p16, and cyclin D2 expression levels of HT-29 and HT-29 CSCs. Scale bar, 20 μm. Protein expressions were assessed using a histopathological scoring system. The asterisks (*) represent significant (*p < 0.05, ***p < 0.001) differences between HT-29 and HT-29 CSCs. (A) Low-expression and mid-expression p53 protein was statistically higher in the HT-29 group (***p < 0.001) than HT-29 CSCs, while its high expression was higher in the HT-29 CSC group (***p < 0.001). (B) Low-expression p21 protein was statistically higher in the HT-29 CSC group (*p < 0.05) than HT-29. p21 protein was higher in the mid-expression HT-29 group (***p < 0.001), while it was elevated in high-expression p21 protein in HT-29 CSCs (***p < 0.001). (C) Low-expression and mid-expression p16 protein was statistically higher in the HT-29 group (***p < 0.001) than HT-29 CSCs (***p < 0.001), while its high expression was higher in the HT-29 CSC group (***p < 0.001). (D) Low-expression cyclin D2 protein was statistically higher in the HT-29 group (***p < 0.001) than HT-29 CSCs, while its mid expression and high expression were higher in the HT-29 CSC group (***p < 0.001).

We compared p53, p21, p16, and cyclin D2 protein levels in HT-29 cells and HT-29 CSCs in our research. The findings for p53 show the low-expression (15.0913 ± 0.72648; 6.7929 ± 0.65769), mid-expression (55.9263 ± 1.03404; 17.3771 ± 1.70989), and high-expression (28.9823 ± 0.95514; 75.8300 ± 1.63382) levels of cells in HT-29 and HT-29 CSCs, respectively. High expression of p53 protein was higher in the HT-29 CSC group (Figure 5A).

The assesment of p21 protein expression showed low-expression (9.6642 ± 0.64885; 14.1601 ± 1.49238), mid-expression (49.8529 ± 1.34485; 16.5748 ± 1.62808), and high-expression (40.4829 ± 1.31125; 69.2651 ± 2.00051) levels of cells in HT-29 and HT-29 CSCs, respectively. High expression of p21 protein was higher in the HT-29 CSC group (Figure 5B).

p16 protein expression was seen as low-expression (22.6460 ± 1.12494; 0.4902 ± 0.49020), mid-expression (47.8372 ± 1.17073; 17.2199 ± 1.55641), and high-expression (29.5168 ± 0.69674; 82.2900 ± 1.79584) levels of cells in HT-29 and HT-29 CSCs, respectively. High expression of p16 protein was higher in the HT-29 CSC group (Figure 5C).

Cyclin D2 results show the low-expression (80.1903 ± 0.73689; 66.1276 ± 0.85541), mid-expression (13.6680 ± 0.86686; 22.7102 ± 0.76147), and high-expression (6.1417 ± 0.35535; 11.1622 ± 0.54064) levels of cells in HT-29 and HT-29 CSCs, respectively. High expression of cyclin D2 protein was higher in the HT-29 CSC group (Figure 5D).

The increased expression of p53, p21, p16, and cyclin D2 proteins in CSCs indicates that the checkpoint is more likely to be dysregulated in these cells, emphasizing their CSC characteristics.

**Figure 6:**
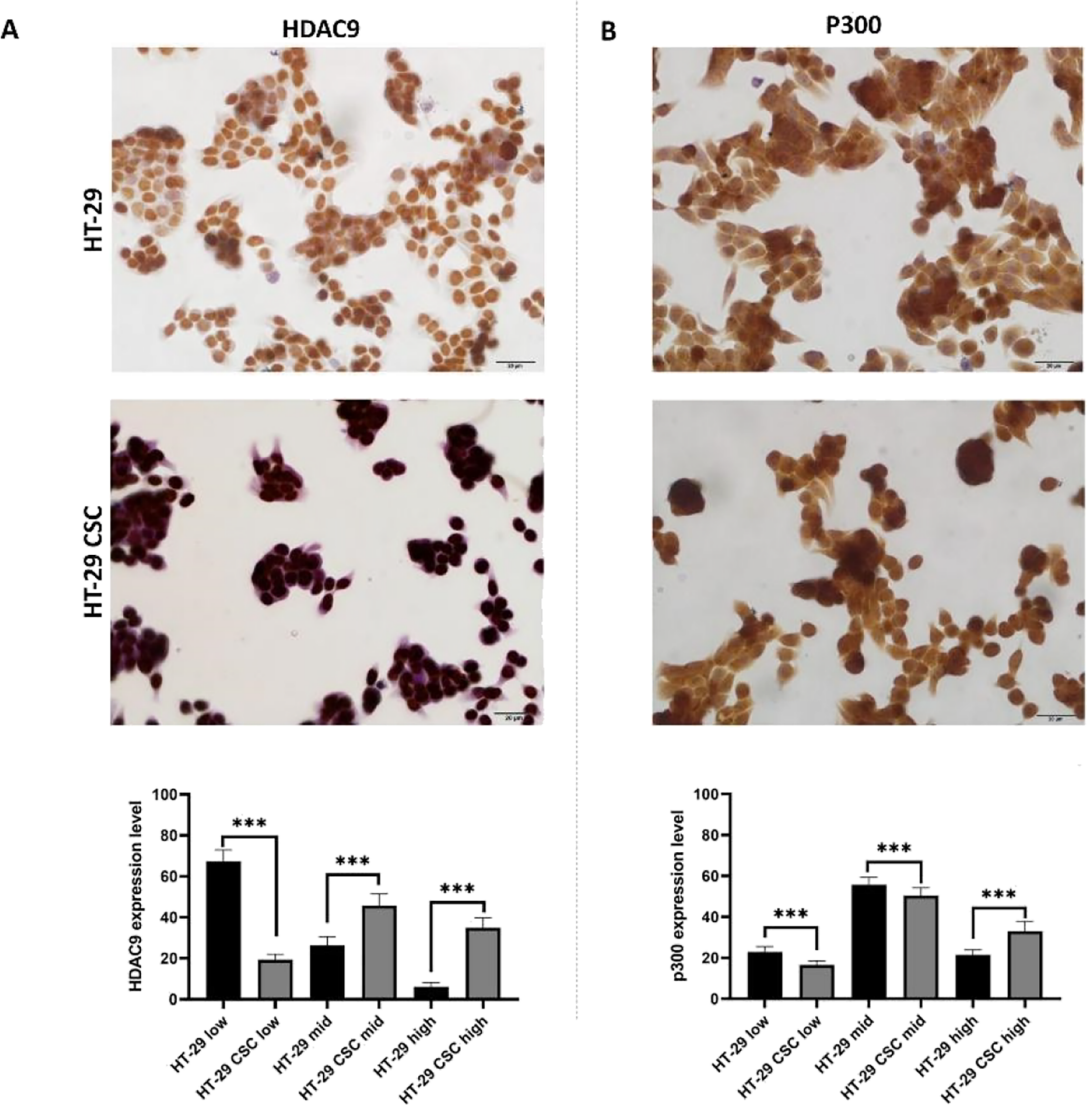
Immunocytochemistry images and graphs of HDAC9 and P300 expression levels of HT-29 and HT-29 CSCs. Scale bar, 20 μm. Protein expressions were assessed using a histopathological scoring system. The asterisks (*) represent significant (***p < 0.001) differences between HT-29 and HT-29 CSCs. (A) Low expression of HDAC9 protein was statistically higher in the HT-29 group (***p < 0.001) than HT-29 CSCs, while its mid expression and high expression were higher in the HT-29 CSC group (***p < 0.001). (B) Low-expression and mid-expression P300 protein was statistically higher in the HT-29 group (***p < 0.001) than HT-29 CSCs (***p < 0.001), while its high expression was higher in the HT-29 CSC group (***p < 0.001).

We compared HDAC9 and P300 protein levels in HT-29 cells and HT-29 CSCs in our research. The findings for HDAC9 show the low-expression (67.3906 ± 1.68316; 19.3096 ± 0.79542), mid-expression (26.3625 ± 1.25335; 45.7326 ± 1.64152), and high-expression (6.2469 ± 0.56372; 34.9578 ± 1.36492) levels of cells in HT-29 and HT-29 CSCs, respectively. High expression of HDAC9 protein was higher in the HT-29 CSC group (Figure 6A).

P300 results show the low-expression (22.8784 ± 0.84126; 16.5712 ± 0.66998), mid-expression (55.6964 ± 1.13770; 50.3717 ± 1.27065), and high-expression (21.4251 ± 0.78106; 33.0571 ± 1.59844) levels of cells in HT-29 and HT-29 CSCs, respectively. High expression of P300 protein was higher in the HT-29 CSC group (Figure 6B).

The elevated levels of epigenetic marker expression in CSCs point to their critical function in preserving CSC features.

**Figure 7:**
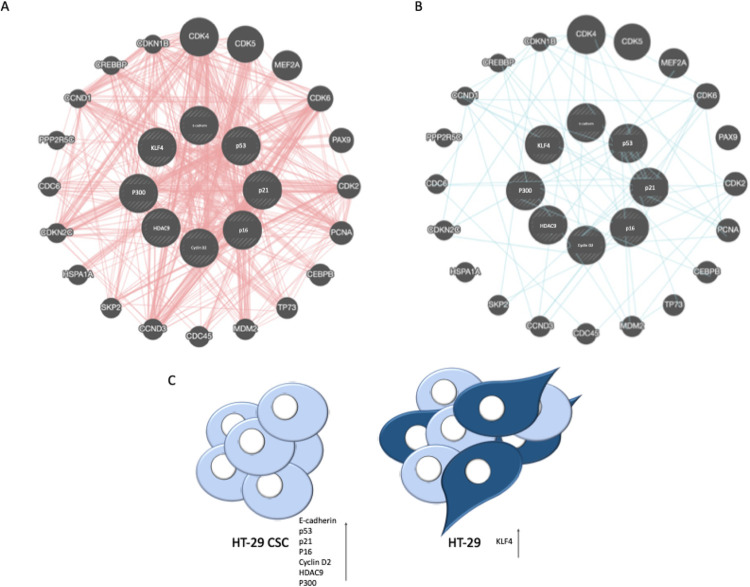
Schematic representation of selected markers with (A) physical interactions with other genes and (B) pathways generated by GeneMania. (C) This schematic diagram reveals foremost markers related with E-cadherin, KLF4, p53, p21, p16, cyclin D2, HDAC9, and P300. According to our results, only KLF4 high level expression was elevated in HT-29 while others in the HT-29 CSC group.

Protein expression differences between cell groups are schematized. Consequently, the findings revealed that, with the exception of the KLF4 protein, the protein expressions were higher in CSCs than HT-29 cells (Figure 7).

## 4 Discussion

Cancers are diverse groups of cells at various stages of differentiation, and abnormal differentiation, which is directed by a stem cell population, could result in altered expression of differentiation markers.^33^ Several studies have revealed that CSCs have a metabolic mechanism distinct from non-CSCs, making them a prospective target for treatment.^34^

For CRC, prompt diagnosis is critical since it can significantly decrease the likelihood of cancer relapse and enhance the survival rate. Although many patients are disease-free following primary tumor treatment, approximately 50% relapse within the first five years after tumor removal.^35,36^ This is caused by presence of CSCs, which are distinguished by treatment resistance, ability to self-renovate, and slow and asymmetric division.^36,37^

CSCs and cancer cells exhibit differences in gene expression related to cellular mechanisms such as regulating the cell cycle, DNA repair pathways, and drug processing.^38,39^ CSC isolation and characterization using different techniques are essential to generating successful CSC treatment regimens. Previous research studies have reported that certain subpopulations in colon cancer express distinct surface biomarkers, including CD133, CD44, and CD26, which are indicative of stem cell-like properties.^5,40,41^ To isolate colon CSCs in our study, we used CD133 and CD44 surface antigens, which have previously been characterized as reliable markers for identifying CSCs.^31,42^

CD133^+^ and CD44^+^ subpopulations have been identified in multiple studies as highly invasive and capable of promoting tumor metastasis.^43−45^ The metastasis is linked to cell migration caused by modifications in the cell skeleton. The scratch assay is useful for researching wound healing mechanisms and the roles of different cell types in the repair process and allows us to compare the migration rate of cells between groups.^46^ A study on HT-29 CSCs showed that triptolide reduces CSC properties and migration rates.^31^ Here, we compared the scratch areas of the HT-29 and HT-29 CSC groups after 72 h of incubation. We normalized the area of scratch according the opening at day 0 and compared the areas day by day. The closure is only statistically different between 0 and 72 h in HT-29 and 0 and 48 and 0 and 72 in HT-29 CSCs. The findings of our study with CRC cells revealed that presence of CD133^+^ and CD44^+^ subpopulations may be associated with metastatic features.

The main factor in cancer patients’ deaths is metastasis. It has been postulated that invasion and metastasis begin following the loss of the intercellular adhesion protein, E-cadherin.^47^ E-cadherin has long been recognized to have a tumor-suppressing function, but there is evidence that it may also have a tumor-promoting function. E-cadherin is expressed in many carcinomas.^48^ Although cadherins are well-established to have important functions in stem cell maintenance, the role of E-cadherin in CRC CSCs is not clear. Previously, two distinct CSC groups were identified according to E-cadherin expression in the primary tumor for the first time. Within the EpCAM^high^/CD44^+^ colorectal CSC population, there were E-cadherin^+^ and E-cadherin^–^ CSCs.^49,50^ Another study discovered that when E-cadherin was reduced in CRC cells, the cells showed a mesenchymal morphology and an elevated CSC marker expression in the colon.^51^ Based on our findings, we found out that HT-29 CSCs had elevated E-cadherin expression, implying that E-cadherin may play a role in carcinogenesis.

The emergence of CSCs is associated with EMT, which promotes cell pluripotency and protects them from chemotherapy by enabling CSCs to persist in the tumor microenvironment.^52^ Significant progress has been made in the CRC CSCs over the past decade, with an increase in the understanding of pluripotency markers. KLF4 is a known pluripotency marker in embryonic development; however, its role in cancer is still unclear. According to the cellular origin and type of cancer, KLF4 can exhibit either tumor suppressor or oncogenic properties. It promotes tumor survival and the progression of various cancers by being overexpressed. KLF4, on the other hand, suppresses stemness in some malignancies.^53,54^ KLF4 expression was reported as a critical feature of colon CSCs, and KLF4 knockdown inhibits tumor initiation, chemoresistance, and expression of CSC markers.^55^ Here, the high-expression level of KLF4 is more prominent in HT-29 cells compared to HT-29 CSCs and is associated with an oncogenic function. Despite the lack of understanding regarding the mechanisms causing these differences, available evidence suggests that KLF4 is a crucial factor in cancer and emphasizes the necessity of further elucidating its specific role in CSCs.

The morphological profile of a cell represents its identity and gives important control over cellular activity. As a result, the morphology of a cell can be thought of as the key to the system that determines cellular fate and functions.^56^ Ultrastructural protrusions on the cell surface play important roles in cancer cell biology because they affect cell motility, migration, cell–cell interaction, and communication. Filopodia, which are cytoplasmic extensions of cells, can be recognized using light microscopy as only their broad end protrudes from the plasma membrane.^57,58^ In addition to light microscopy techniques, SEM has been extensively used in cell culture systems to offer higher resolution and three-dimensional visualization of cell surface morphology.^57,59^ Previous research has demonstrated that mouse embryonic stem cells (mESC), cancer cells, and somatic cells exhibit distinct morphologies, with mESCs being round, cancer cells being highly irregular, and somatic cells being elongated and spindle-shaped.^56^ In our study, we revealed the ultrastructural level of the presence and morphological characteristics of cytoplasmic protrusions, namely, filopodia, in HT-29 cells and HT-29 CSCs. SEM images of these cells show numerous filopodia with differing lengths. The number and length of these long and thin filopodia were similar in the cells of both groups. In addition, both cells have irregular cell borders. The cell surface of HT-29 CSCs appears to be uneven, with some areas appearing smooth, while others exhibit roughness. These cells exhibit a rougher surface, particularly in their peripheral areas, as compared to HT-29 cells.

Based on the region of interest under examination, SEM paired with EDS determines the elemental composition. There has been limited research into SEM/EDS, which reveals the elemental composition of a region of interest.^56,60^ The ultrastructures of both HT-29 cells and HT-29 CSCs were thoroughly documented and investigated using SEM/EDS in the current work. Elemental components were classified and analyzed based on their respective locations within the cells. HT-29 cells and HT-29 CSCs were compared by SEM/EDS, and six different elemental signals were obtained. The elements within cells were C, N, O, Na, P, and K, which were similar with the research that detects P, Na, O, Ca, and Mg within the thyroid psammoma bodies.^61^ In the EDS spectrum of all cell groups, the percentage of carbon (C) in the central areas was higher than in the peripheral areas, while sodium (Na) was low. The percentage of oxygen (O) was found to be higher in the HT-29 CSCs’ peripheral areas. HT-29 and HT-29 CSCs had nuclear (central) and peripheral areas, and several spots from these areas were examined for elemental ratio comparison. The results were consistent in terms of C, Na, and O values in papillary thyroid carcinoma (PTC) cells in the previous research comparing the normal thyroid cell line NTC and PTC.^62^

One of the prominent strategies in cancer treatment is the cell cycle regulation system, specifically checkpoints. The period and the timing of the cell cycle phase transitions fluctuate significantly between cell types.^63,64^ The ability of CSCs to be dormant or quiescent is an important feature that makes them resistant to standard treatment. CSCs, particularly after treatment, have the ability to enter into a reversible G0 phase and remain dormant.^65^ In human colon cancer, Takaya et al. discovered that CSCs are predominantly found in the G0/G1 cell cycle phase compared to nonstem cell cancer cells.^66^ In our study, we compared cell cycle analyses of HT-29 CSCs and HT-29 cells, which are sources of heterogeneity in colorectal carcinoma, in cell cycle phases. According to our results, we did not find any significant difference between the two groups in G0/G1, S, and M phases. However, we noticed that the most abundant percentage of both cell groups was in the G0/G1 phases after 72 h of incubation.

Increased expression and accumulation of p53, as well as p16 and p21, can cause cell cycle arrest.^67^ These molecular factors are also involved in the apoptotic process.^68^ Particular variations in cell cycle gene expression have been observed between CSCs and the tumor cell population that they reside in, according to several studies.^69,70^

p21’s function is influenced by the cellular and environmental context, with variations observed across different cells. The presence of both tumor suppressor properties and its role in oncogenesis supports this notion. This feature is marked by increased levels of p21 expression in stem cells. p21 blocks cell cycle progression during the G1 and S phases. It has been demonstrated that p21 plays a role in preserving tumor initiation potential in colon CSCs and that p21 expression is higher in CSCs than in non-CSCs.^71^ These outcomes suggest that p21 could have a vital function in stemness. Our findings supported the notion that p21 is linked to cancer stem cells and showed that p21 expression is higher in HT-29 CSCs.

Somatic TP53 mutations are present in up to 60% of CRC patients and are linked to unfavorable clinical consequences.^72^ TP53 is activated and p53 increases in stressed cells, which causes an arrest in the cell cycle.^73^ Mutant p53 is restricted to poorly differentiated tumors, whereas wild-type p53 expression increase is restricted to less differentiated tumor areas. These findings show that mutant p53 is associated with CSC formation and a negative outcome.^74^ By comparing the amounts of CD133^+^ cell populations in the HCT116 p53+/+ and p53–/– groups, researchers discovered in 2018 that p53 regulates the expression of the CSC marker CD133. The research specifically showed that p53 silencing decreased CD133 expression, whereas p53 overexpression increased it.^75^ Our findings supported previous research that linked p53 to stem cell biology and demonstrated that p53 expression was higher in HT-29 CSCs.

p16 is a protein that helps regulate the cell cycle and belongs to a class of proteins called INK4 inhibitors. In cancer, p16 is often not working properly. By binding and inactivating certain proteins called CDK4 and CDK6, p16 helps stop cells from dividing. p16 also interacts with other important genes that help prevent cancer, like retinoblastoma and p53.^76^ The incidence of p16 expression in various cancer types remains a matter of debate. For many tumors, the reported frequency of p16 positivity varies greatly. In colorectal adenocarcinoma, p16-positive cases ranged from 9 to 98%.^77^ Reduced expression and overexpression have both been linked to poor prognosis in CDK 4 and 6 in the G1–S transition of several cancer types.^78,79^ A study using Hela cervical cancer cells demonstrates that p16 shRNA gene silencing resulted in decreased CSC marker expressions and the tumorigenic potential ability of the cells.^80^ Conversely, other studies using breast, pancreas, and Hela cervical cancer cell lines reported that reduced expression of p16 was associated with CSCs.^81−83^ Our study reported that p16 expression was higher in HT-29 CSCs. This is in concordance with Wu et al., who stated a similar result.

Type D cyclins are essential components of the cell cycle, and cyclin D overexpression has been linked to tumor growth in CRC. Cyclin D2 is encoded by CCND2, a protein that regulates CDKs 4 and 6 involved in the G1–S transition and thus contributes to the progression of the cell cycle. While cyclin D2 expression is increased in cancer, there are certain cancer types in which its expression is decreased.^84,85^ Cyclin D2 was found to be overexpressed in 53% of colon malignancies and has been associated with a higher TNM stage of tumors, indicating a possible metastatic function for CCND2.^86^ Park et al. discovered that CSCs had significantly higher CCND2 expression. Silencing CCND2 in CSCs decreased their intrinsic defense systems, leading to cell cycle changes, loss of proliferation potency, and DNA damage accumulation after radiation treatment. Their findings show that CCND2 may play a role in CSC plasticity regulation.^87^ We found that HT-29 CSCs have higher levels of cyclin D2 expression than the HT-29 cell line and that this supports the oncological function in CRC.

The process of tumor development involves multiple factors and stages.^88^ Aberrant epigenetic alterations are one of the defining characteristics of malignancies. Histone acetylation and deacetylation are epigenetic modifications that have been involved in the modulation of stemness characteristics during both normal and tumorigenic activities.

HDAC9 has a complex role in tumorigenesis. This molecule could promote cancer development while could have a tumor suppressive function.^89−91^ In a previous study using prostate cancer cell lines, exogenous overexpression of CD133 was found to upregulate HDAC9 expression. In addition, inhibition of HDAC9 expression led not only to increased expression of E-cadherin but also to decreased migration capacity. These findings suggest that HDAC9 inhibition is a crucial factor in regulating the EMT process in CSC-like cells.^92^ According to our findings, HDAC9, as an oncogene, was much more expressed in HT-29 CSC cells, but it was also expressed in HT-29 cells and was associated with CRC.

P300, a classic endogenous HAT and a tumor-promoting protein, is important for the progression of most solid tumors. P300 has been linked to the critical process of malignant tumor formation biology, including tumor cell proliferation, migration, and invasion.^15^ Increased P300 expression has been linked to aggressive tumor features and poor clinical outcomes in a number of solid tumors.^93−96^ There have been very few studies attempting to define the role of P300 expression in cells with the CSC phenotype. Our study’s findings demonstrate a considerable increase in P300 expression in HT-29 CSCs compared to the HT-29 cell line, suggesting that P300 may play a role in controlling the stemness of carcinogenesis.

## 5 Conclusions

Cancers are heterogeneous populations of cells at various stages of differentiation. Cancer treatment resistance is caused by a variety of factors, through different regulations of signaling pathways in heterogeneous tumor cells. We attempted to reveal the similarities and differences in migration, EMT, pluripotency, morphology, cell division, factors regulating cell division, and epigenetic factors between CSCs and non-CSCs, which are the source of heterogeneity in colorectal cancer. In this current study, we showed that while migration, EMT, cell division, markers regulating cell division, and epigenetic markers were all expressed at high levels in the HT-29 CSC group, KLF4, one pluripotency marker but acts in variable roles in cellular processes, was expressed at high levels in the HT-29 group. Our findings, along with the evaluation of other stem cell-related transcription factors in tumors, such as CRC, will enhance our comprehension of the diverse mechanisms underlying signaling pathway regulation and thus facilitate the identification of cancer-specific CSCs. This could pave the way for the development of biomarker panels for both diagnosis and prognosis, as well as patient-specific tumor surveillance as a proxy for therapeutic response.

## Data Availability

The datasets used and/or analyzed during this study can be received from the corresponding author upon reasonable request.
